# Increase in invasive group A streptococcal infection notifications, England, 2022

**DOI:** 10.2807/1560-7917.ES.2023.28.1.2200942

**Published:** 2023-01-05

**Authors:** Rebecca Guy, Katherine L Henderson, Juliana Coelho, Helen Hughes, Emily L Mason, Sarah M Gerver, Alicia Demirjian, Conall Watson, Ashley Sharp, Colin S Brown, Theresa Lamagni

**Affiliations:** 1 HCAI, Fungal, AMR, AMU and Sepsis Division, UK Health Security Agency, London, United Kingdom; 2 Staphylococcus and Streptococcus Reference Section, AMRHAI, UK Health Security Agency, London, United Kingdom; 3 Real-time Syndromic Surveillance Team, Field Services Division, UK Health Security Agency, London, United Kingdom; 4 Department of Paediatric Infectious Diseases & Immunology, Evelina London Children’s Hospital, London, United Kingdom; 5 Faculty of Life Sciences & Medicine, King’s College London, London, United Kingdom; 6 Immunisation and Vaccine-Preventable Diseases Division, UK Health Security Agency, London, United Kingdom

**Keywords:** Group A streptococcus, invasive, children, England, *Streptococcus pyogenes*

## Abstract

Increases in invasive group A streptococcal (iGAS) infection and associated deaths, particularly in children, above seasonally expected levels are being seen this season (772 notifications reported in weeks 37 to 48 in 2022) across England. Diagnoses of iGAS infection from lower respiratory tract specimens in children under 15 years increased to 28% in November 2022. Medical practitioners have been alerted to the exceptional increase in incidence, including unusual numbers of children presenting with pulmonary empyema.

Following an unusual seasonal pattern of both scarlet fever (caused by group A *Streptococcus*) and invasive group A streptococcal (iGAS) infection in children earlier in 2022 [1,2], with elevated activity continuing until August 2022 (school summer holidays), further increases in incidence are again being seen. Spontaneous reports from clinicians of lower respiratory tract (LRT) iGAS presentations in children younger than 15 years with respiratory virus co-infection increased in November 2022. Using data linkage, we investigated trends in rates of iGAS infection, clinical presentations and respiratory virus co-infection in England, with a focus on children younger than 15 years to align with the case definition implemented by the national incident management team.

## Invasive group A streptococcal infections

**Figure 1:**
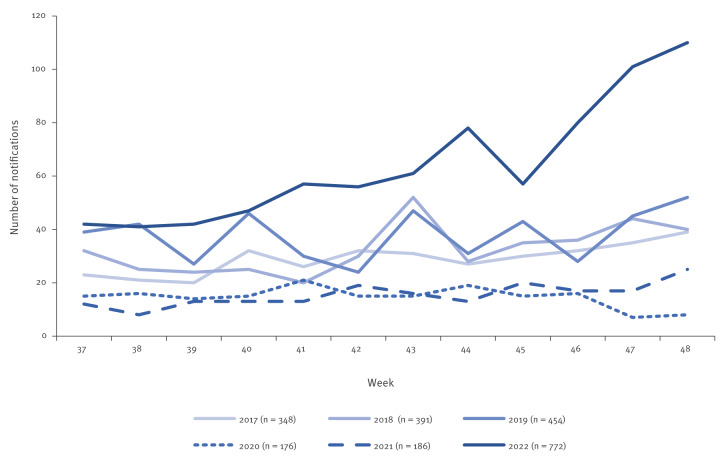
Weekly laboratory notifications of invasive group A streptococcal infections, England, weeks 37–48, 2017–2022^a^ (n = 2,327)

^a^ This figure compares the first 12 weeks of the season, all of which fall into the first year the season covers.

In England and Wales, iGAS infection is statutorily notifiable, with microbiologically confirmed diagnoses subject to urgent notification (within 24 h) to facilitate contact follow-up as per national guidance [3,4]. Local laboratories and the national reference laboratory report data on iGAS infection (diagnosed through culture or using molecular methods, including rapid strep antigen testing) in England to the United Kingdom Health Security Agency (UKHSA) Second Generation Surveillance System (SGSS). Laboratory notifications of iGAS infection in the first 12 weeks (weeks 37 to 48) of the 2022/23 season (a season runs from week 37 one year to 36 the next; mid-September to mid-September) showed week-on-week elevations, above levels expected for this time of year in England. Marked increases occurred particularly in weeks 46 to 48. A total of 772 notifications were reported between weeks 37 and 48 of 2022, with 110 cases notified in week 48 (Figure 1). Cases were regionally dispersed with increases seen across England.

At the time of analysis, the age distribution of iGAS cases in England for the 2022/23 season had shifted from previous seasons, with a substantial drop in median age to 46 years (range: 0–102), lower than in the five previous seasons (medians of 53 to 57 years). Pronounced increases in incidence were recorded in children, with a quarter (26.1%; 202/772) of all iGAS infections diagnosed in children younger than 15 years in 2022/23, compared with a range of 6.4–13.3% in the previous seasons. Rates of iGAS infection were elevated compared with the same point in the previous five seasons for children younger than 1 year (2.5 vs 0.2–1.3/100,000), 1–4-year-olds (4.1 vs 0.1–1.3/100,000) and 5–9-year-olds (1.8 vs 0.1–0.6/100,000). In 2022/23, for cases where sex was available, 57% (433/758) of all iGAS cases were male and 43% female (325/758). Of the cases younger than 15 years, 63% (125/198) were male and 37% were female (73/198). Sex was unavailable for 14 iGAS cases, four of whom were in the age group under 15 years.

**Figure 2:**
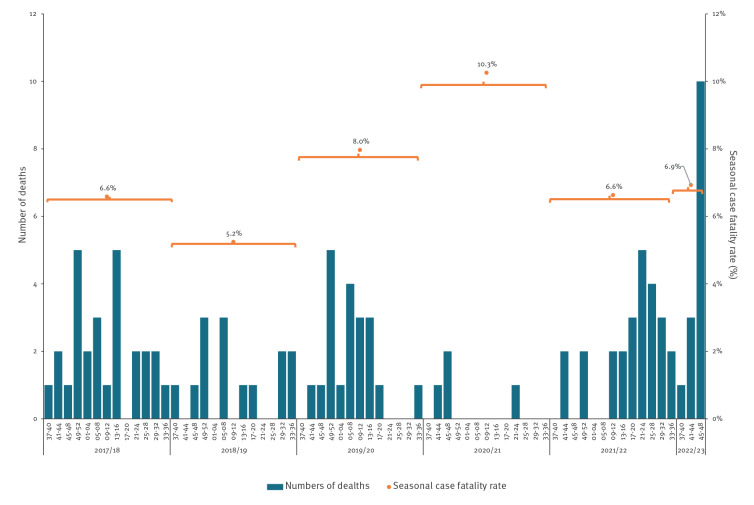
Seven-day all-cause mortality following diagnosis of invasive group A streptococcal infection and seasonal case fatality rate^a^ in children younger than 15 years, England, week 37/2017–week 48/2022 (n = 104)

^a^ Mortality during the coronavirus disease pandemic period should be interpreted with caution as few episodes of invasive group A streptococcal infection were recorded across this period.

With the increase in absolute numbers of iGAS cases in children, an increase in numbers of deaths has been seen; 14 deaths (7-day all-cause mortality) in children younger than 15 years were reported from week 37 to 48 in 2022 (Figure 2), accounting for 14 of all 61 iGAS deaths in all age groups during this period. Between four and 27 deaths within 7 days of iGAS infection occurred in this age group in each full season in the previous 5 years. The 7-day all-cause case fatality rate (CFR) in 2022 for children younger than 15 years was within range of past seasons (6.9%; 95% confidence interval (CI): 3.8–11.4 vs 2018/19 with 5.2%; 95% CI: 2.9–8.6 and 2020/21 with 10.3%; 95% CI: 2.9–24.2), and was similarly within range for children under 10 years (5.3%; 95% CI: 2.6–9.6 vs 2018/19 with 4.9%; 95% CI: 2.6–8.4 and 2019/20 with 7.3%; 95% CI: 4.3–11.4). The CFR in other age groups have remained within expected ranges compared with previous years. 

The orange brackets indicate that the case fatality rate applies to the entire season.

## Distribution of invasive group A streptococcal specimen types in age groups over time

**Figure 3:**
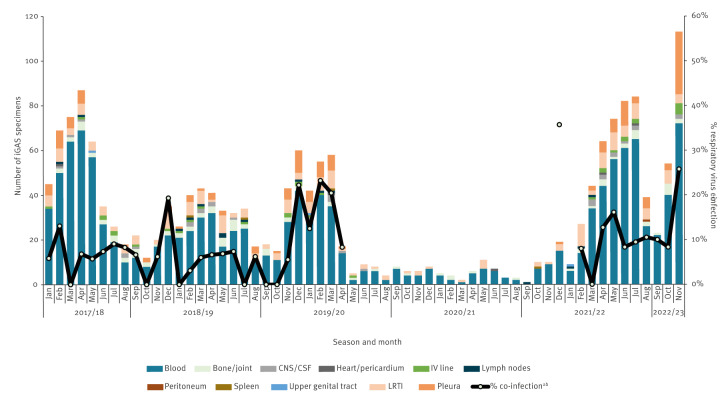
Monthly number of iGAS infections in < 15-year-olds by specimen type and percentage with respiratory virus co-infection^ab^ (within +/− 1 day), England, January 2018^c^–November 2022 (n = 1,830)

CNS: central nervous system; CSF: cerebrospinal fluid; iGAS: invasive group A *Streptococcus*; IV: intravascular; LRTI: lower respiratory tract infection.

^a^ Where fewer than 10 iGAS were recorded in a month, the co-infection percentage is not presented.

^b^ A patient can have more than one invasive specimen type reported and could be included multiple times in this graph. For children < 15 years, the total number of invasive specimens (n = 113) does not equate to the total number iGAS infections (n = 97).

^c^ 2017/18 is a partial season; due to data availability, specimen numbers for this season are only presented for the period January to August 2018.

Laboratory notifications of iGAS infection (sterile site specimens) are reported to UKHSA SGSS with information on the specimen type, potentially including multiple specimens per patient. Data presented in this section describe the individual specimen types reported for iGAS infections. Specimen type data were extracted for January 2018 to November 2022, with each specimen type counted as a separate specimen episode. Historically, laboratory notifications of iGAS infections have predominantly been reported from blood culture specimens; since 2018, an average 87% of monthly reports have been from blood specimens and 5% from pleura/LRT specimens (17% in < 15-year-olds). However, during this early part of the 2022/23 season, iGAS infections diagnosed from LRT specimens including pleural fluid specimens increased (12% of iGAS specimens in November 2022 for all ages; 44/365), particularly in children younger than 15 years (28% in November; 32/113) (Figure 3). Similar increases in pleura/LRT specimens were not noted in the older age groups.

## Respiratory virus coinfections

Respiratory viral infections are reported into UKHSA SGSS or the sentinel surveillance respiratory virus DataMart. Data presented in this analysis are patient-level laboratory-confirmed diagnoses made in England [5]. 

Increased reports of iGAS cases in children younger than 15 years with respiratory virus co-infections (+/− 1 day of the iGAS specimen) were noted in October and November 2022. During November 2022, the latest complete month with available data, 25.8% (25/97) of < 15-year-olds with iGAS had a recorded co-infection with a respiratory virus within +/− 1 day, similar to levels seen in past seasons at this time of year (Figure 3). The most frequently identified co-infecting respiratory viral species were respiratory syncytial virus (RSV) (n = 12; 12.4% iGAS cases in November 2022), human metapneumovirus (hMPV) (n = 11; 11.3%) and rhinovirus (n = 7; 7.2%) (Table). This is in line with increasing numbers of RSV and rhinovirus infections detected in England since week 37/2022 [6]. Of the 10 deaths in children younger than 15 years (7-day all-cause mortality) diagnosed with iGAS infection in November 2022, five were identified as having a respiratory virus co-infection.

**Table:** Respiratory viral co-infections within +/− 1 day of invasive group A streptococcal infection, by age group, in children younger than 15 years, England, November 2022 (n = 97)

	Age group				Total
	< 1 year	1–4 years	5–9 years	10–14 years	
Number of children with iGAS	10	50	30	7	97
Number of children with co-infections	3	15	6	1	25
**Co-infections^a^**	**5**	**32**	**11**	**1**	**49**
Adenovirus	0	2	1	0	3
Severe acute respiratory syndrome coronavirus 2	1	3	1	0	5
Enterovirus	0	3	0	0	3
Human metapneumovirus	1	8	2	0	11
Influenza A	1	0	1	1	3
Influenza B	0	1	0	0	1
Parainfluenza	0	3	1	0	4
Respiratory syncytial virus	2	8	2	0	12
Rhinovirus	0	4	3	0	7

Seasonal coronaviruses were also included in the analysis, with none involved in an invasive group A streptococcal co-infection.

^a^ Some children (n = 97) had more than one respiratory viral pathogen at the same time; each of these are counted separately in this total.

## *emm* types

Typing data were available for 80% (617/772) of iGAS isolates (from all age groups) from this season. No unusual *emm* types have been detected. The main *emm* types in those 15 years and older were *emm* 1 (31%), *emm* 12 (16%), *emm* 89 (6%) and *emm* 33 (5%). In children younger than 15 years, *emm* 1 was the main (57%) circulating *emm* type, followed by *emm* 12 (23%) and *emm* 4 (7%) (the detailed numbers are listed in the Supplement). Typing results have been reported for 13 child deaths, of which nine were *emm* 1, three were *emm* 12 and one was *emm* 77. Whole genome sequencing of all typed invasive and non-invasive isolates indicates that there has been no obvious expansion of a single clone for those emm types, however, the majority of the *emm* 1 isolates were part of the *emm* 1 M1UK clone, first detected in 2010, for which upregulation of SpeA expression, a virulence factor of *Streptococcus pyogenes*, has been demonstrated [7,8]. 

## Scarlet fever

In England and Wales, statutory notifications of scarlet fever, based on clinical symptoms, are submitted by diagnosing clinicians to local public health officials. General practitioner (GP) consultations for scarlet fever and sore throat, as well as notifications of clinically diagnosed scarlet fever also showed marked elevations, with a total of 8,688 notifications made between week 37/2022 and week 48/2022, compared with 333–2,536 notifications in the same period of the previous seasons 2017/18 to 2021/22 [1,5]. A total of 2,711 notifications were received across England in week 48 of 2022.

## Discussion

The United Kingdom (UK) is experiencing a surge of iGAS infections, particularly in those younger than 15 years, which is of considerable concern given the associated risk of poor outcome and the added pressure on emergency departments and paediatric intensive care units [9]. A commensurate increase in number of deaths in children with iGAS infection has been seen.

Nationwide alerts were issued to frontline clinicians on 2 December 2022, including a request for identification of routine surveillance reports relating to lower respiratory tract iGAS presentation in children younger than 15 years. Similar concerns about increases in paediatric iGAS cases are also being reported in the Netherlands, France, Ireland and the United States [10-14]. In the Netherlands, the increase seen earlier in 2022 was particularly apparent in children younger than 5 years, with *emm* types similar to the ones in England for all ages (*emm* 1, 12 and 4); high levels of chickenpox activity, also noted in England [1,2], were attributed to the rise in iGAS cases [10]. The multinational nature of current signals suggests that this phenomenon may relate to decreased exposure to group A streptococci and other childhood infections during the coronavirus disease (COVID-19) pandemic. Measures such as physical distancing and face masks introduced during the COVID-19 pandemic are likely to have reduced exposure to a range of common childhood infections, which may subsequently have impacted childhood immunity [15].

Given the high levels of respiratory virus co-infection in children presenting with iGAS infection, parents of children with presumed respiratory viral infection are being made aware through public health messaging of features suggestive of secondary bacterial infection, including clinical deterioration, and when and how to seek further help. The UKHSA has asked laboratories in England to forward all cultured group A streptococcal isolates associated with empyema and other lower respiratory tract infections in children for typing as continued vigilance for the emergence of a novel strain or changes in pattern of clinical disease is essential. Further, UKHSA has alerted medical practitioners to this early increase in incidence and elevated iGAS infection in children, with further increases seen in December 2022 [16]. Prompt treatment of scarlet fever cases with antibiotics remains important to limit further spread and reduce risk of potential complications in cases and their close contacts [17].

## Conclusion

In light of the increase in iGAS infections being seen in the UK and other countries, close monitoring and awareness by clinicians and the wider public remains a critical public health measure. Prompt notification of iGAS cases for assessment of close contacts to identify those in need of prophylactic treatment is important, as well as timely initiation of antibiotic treatment for scarlet fever and other group A streptococcal presentations to reduce risk of complications and limit onward transmission. 

## Supplementary Data

22000 Supplement
